# The management of active surveillance in prostate cancer: validation of the Canary Prostate Active Surveillance Study risk calculator with the Spanish Urological Association Registry

**DOI:** 10.18632/oncotarget.21984

**Published:** 2017-10-24

**Authors:** Ángel Borque-Fernando, José Rubio-Briones, Luis Mariano Esteban, Argimiro Collado-Serra, Yoni Pallás-Costa, Pedro Ángel López-González, Jorge Huguet-Pérez, José Ignacio Sanz-Vélez, Jesús Manuel Gil-Fabra, Enrique Gómez-Gómez, Cristina Quicios-Dorado, Lluis Fumadó, Sara Martínez-Breijo, Juan Soto-Villalba

**Affiliations:** ^1^ Department of Urology, Hospital Universitario Miguel Servet, IIS-Aragón, Zaragoza, Spain; ^2^ Department of Urology, Instituto Valenciano de Oncología, Valencia, Spain; ^3^ Escuela Universitaria Politécnica de La Almunia, Universidad de Zaragoza, Zaragoza, Spain; ^4^ Department of Urology, Hospital de Manises, Valencia, Spain; ^5^ Department of Urology, Hospital Clínico Universitario Virgen de la Arrixaca, Murcia, Spain; ^6^ Department of Urology, Hospital Clinic de Barcelona, Barcelona, Spain; ^7^ Department of Urology, Hospital General San Jorge, Huesca, Spain; ^8^ Department of Urology, Hospital Universitario Reina Sofía, IMIBIC, Córdoba, Spain; ^9^ Department of Urology, Fundación Jimenez Díaz, Madrid, Spain; ^10^ Department of Urology, Hospital del Mar, Barcelona, Spain; ^11^ Department of Urology, Complexo Hospitalario Universitario A Coruña, A Coruña, Spain; ^12^ Department of Urology, Hospital Universitario Puerta del Mar, Cádiz, Spain

**Keywords:** active surveillance, prostate cancer, reclassification, risk calculator, external validation

## Abstract

The follow up of patients on active surveillance requires to repeat prostate biopsies. Predictive models that identify patients at low risk of progression or reclassification are essential to reduce the number of unnecessary biopsies. The aim of this study is to validate the Prostate Active Surveillance Study risk calculator (PASS-RC) in the multicentric Spanish Urological Association Registry of patients on active surveillance (AS), from common clinical practice.

**Results:**

We find significant differences in age, PSA and clinical stage between our validation cohort and the PASS-RC generation cohort (*p* < .0001), with a reclassification rate of 10–22% on the follow-up Bx, no cancer was found in 43% of the first follow-up Bx. The calibration curve shows underestimation of real appearance of reclassification. The AUC is 0.65 (C.I.95%: 0.60–0.71). PDF and CUC do not suggest a specific cut-off point of clinical use.

**Methods:**

We select 498 patients on AS with a minimum of one follow-up biopsy (Bx) from the 1,024 males registered by 36 Spanish centers recruiting patients on the Spanish Urological Association Registry on AS. PASS-RC external validation is carried by means of calibration curve and area under de ROC-curve (AUC), identifying cut-offs of clinical utility by probability density functions (PDF) and clinical utility curves (CUC).

**Conclusions:**

In our first external validation of the PASS-RC we have obtained a moderate discrimination ability, although we cannot recommend cut-off points of clinical use. We suggest the exploration of new biomarkers and/or morpho-functional parameters from multiparametric magnetic resonance image, to improve those necessary tools on AS.

## INTRODUCTION

Active surveillance (AS) is increasingly being implemented by urologists as a strategy that provides the benefits from prostate cancer (PCa) opportunistic screening observed in western countries [1], but avoids overtreatment. We have presented our National Registry (AEU/PIEM/2014/0001, www.piem.aeu.es) supported by the Spanish Urological Association (Asociación Española de Urología, AEU) [2]. This initiative was created with the aim of facilitating the implementation of AS in all types of Hospitals, and of providing an opportunity for multicentric clinical research, as different inclusion criteria and follow-up strategies are allowed. These wider inclusion criteria are contemplated in other AS registers such as the Canary Prostate Active Surveillance Study (PASS) [3], in contrast with the more strict inclusion criteria used by previous series [4–10]**.**

All the different AS protocols coincide on the necessity to perform repeated prostatic biopsies (Bx) which are not free of complications [11], with increasing antibiotic resistance as a major problem. Recently, the initial results of the PASS study (clinicaltrials.gov NCT00756665) have been published, with a median follow-up of 28 months: 24% of their patients faced adverse reclassification [3]. These data are frequently found in a current database like ours [2] among others [12, 13], all of which have been previously driven by the uncertainty of the common selection criteria used. Using a cohort of 859 Gleason 6 PCa patients, in their late study the same group has proposed an easy to use online prediction tool [14] of progression in AS, with widely used and reproducible covariates. This tool showed an area under the curve (AUC) of 72.4% for reclassification in the follow-up Bx, providing an on-line tool to facilitate counselling for the patients on AS [15].

Although new biomarkers and data from multiparametric Magnetic Resonance Imaging (mpMRI) could improve its predictive accuracy [16–19], the real strength of this new tool relies on its variables: age, PSA, months from last biopsy, percentage of positive cores for PCa on the last Bx and number of prior negative Bx; all of them available and reproducible elsewhere. Both criteria, new biomarkers such as PCA3 and PHI [20] and mpMRI [21], have shown their ability for a better selection of patients for AS when referred to pathological results of radical prostatectomy specimens, but they will need to demonstrate clear advantages in gaining accuracy, but also their cost-effectiveness, if they are to be regularly introduced in AS protocols. Without mpMRI and new biomarkers and using just the criteria utilized in this model, the long term results of the AS series show excellent disease-free survival results [19].

As the PASS and AEU protocols are comparable in their design [2, 3], we aim to perform an external validation of this PASS-RC with a different population. We understand that our external validation study specially focused on defining clinical utility of PASS-RC could make this tool a useful help for decision making in men on AS.

## RESULTS

From the 1,024 Prostate cancer patients recruited in the PIEM cohort until December 31st 2015, only 498 of them had at least one follow-up Bx to evaluate the possibility of Bx reclassification by the PASS-RC. These 498 patients form the validation set and come from 24 Spanish hospitals, the median follow-up was 19.7 months with 25%–75% percentiles of 11.4 and 33.7 months, respectively. Among them, the patients without reclassification had a median follow-up until last biopsy of 11.7 months, with P25–75 of 7.2–18.3. The median time between reclassification and first biopsy was 9.5 months with P25–75 of 7–16.7 months. We observed statistically significant differences for the distribution of variables age, race, PSA and T stage at diagnosis between both cohorts (Table 1).

**Table 1:** Descriptive characteristics of PASS cohort versus PIEM-AEU- AS validation series

Variable		PIEM-AEU-AS (*N* = 498)		PASS (*N* = 859)		*p*-value
		*N*	%	*N*	%	
Age at diagnosis						
	≤49	5	1,00%	40	4,66%	<0,001
	50–59	102	20,48%	251	29,22%	
	60–69	248	49,80%	461	53,67%	
	70–79	143	28,71%	106	12,34%	
	≥80	0	0,00%	1	0,12%	
Race						
	White	460	92,37%	783	91,15%	<0,001
	Black	1	0,20%	43	5,01%	
	Other-NA	37	7,43%	33	3,84%	
PSA at diagnosis, ng/mL						
	0–2.5	11	2,21%	104	12,11%	<0,001
	2.5–4	83	16,67%	148	17,23%	
	4–6	193	38,76%	352	40,98%	
	6–10	176	35,34%	192	22,35%	
	>10	35	7,03%	53	6,17%	
	NA	0	0,00%	10	1,16%	
T stage at diagnosis						
	T1a-c	466	93,57%	763	88,82%	<0,001
	T2a	27	5,42%	92	10,71%	
	T2b-c	1	0,20%	4	0,47%	
	Na	4	0,80%	0	0,00%	

Our cohort had a maximum of four follow-up Bx (Table 2), with slightly superior patient mean age at the different biopsies. The follow-up Bx had been performed at a mean of 2 months before in the AEU cohort. The mean PSA value was approximately 1 ng/ml greater in our series. The number of biopsy cores had the same mean 12 cores for the diagnostic biopsy at both series, but our validation cohort practiced significantly more cores at the follow-up-Bx.

**Table 2:** Biopsy characteristics at diagnosis and at each sequential surveillance biopsy

Characteristics					Biopsy					
	Diagnosis		First		Second		Third		Fourth	
	PIEM	PASS	PIEM	PASS	PIEM	PASS	PIEM	PASS	PIEM	PASS
Patients, *n*	1024	979	498	859	100	458	20	211	9	75
Age at biopsy, yr, mean (SD)	66.1 (6.9)	62.0 (6.9)	66.5 (7.0)	63.0 (7.0)	66.4 (6.8)	64.4 (6.9)	66.2 (6.1)	65.5 (6.9)	67.1 (4.8)	65.5 (7.2)
Months since last biopsy, no, mean (SD)	0 (0.0)	0 (0.0)	10.9 (8.9)	12.8 (8.8)	18.4 (8.5)	19.7 (8.3)	18.9 (8.5)	21.1 (7.9)	18.0 (10.0)	20.1 (7.8)
Most recent PSA, ng/ml, mean (SD)	6.6 (3.0)	5.5 (3.0)	6.2 (3.5)	5.0 (3.3)	6.3 (3.6)	5.2 (3.6)	6.1 (4.3)	5.4 (3.6)	8.8 (6.1)	5.5 (3.9)
No. of biopsy cores, median (range)	12 (10–70)	12 (4–60)	16 (10–67)	12 (4–60)	16 (10–31)	12 (4–46)	18 (10–24)	12 (4–46)	20 (12–27)	12 (4–34)
Percentage of cores positives for cancer, *n* (%)										
0	0 (0.0)	0 (0.0)	218 (43.8)	312 (36.3)	49 (49)	190 (41.5)	10 (50)	88 (41.7)	4 (44.45)	30 (40.0)
>0 and <34	1014 (99.0)	860 (87.8)	251 (50.4)	465 (54.1)	44 (44)	224 (48.9)	8 (40)	(49.3)	4 (44.45)	41 (54.7)
≥34	6 (0.6)	37 (3.8)	29 (5.8)	66 (7.7)	7 (7)	37 (8.1)	2 (10)	18 (8.5)	1 (11.1)	4 (5.3)
NA	4 (0.4)	82 (8.4)	—	16 (1.9)	—	7 (1.5)	—	1 (0.5)	—	0 (0.0)
Gleason score, n (%)										
≤6	1024 (100)	979 (100.0)	413 (82.9)	732 (85.2)	83 (83)	384 (83.8)	19 (95)	166 (78.7)	7 (77.8)	63 (84.0)
7	0 (0)	0 (0.0)	76 (15.3)	123 (14.3)	13 (13)	70 (15.3)	1 (5)	43 (20.4)	2 (22.2)	10 (13.3)
≥8	0 (0)	0 (0.0)	9 (1.8)	4 (0.5)	4 (4)	4 (0.9)	0 ()	2 (1.0)	0 ()	2 (2.7)
Outcome										
Reclassification	0 (0)	0 (0.0)	94 (18.9)	163 (19.0)	17 (17)	104 (22.7)	2 (10)	61 (28.9)	2 (22.2)	19 (25.3)
Stable	1024 (100)	897 (91.6)	404 (81.1)	684 (79.6)	83 (83)	347 (75.8)	18 (90)	149 (70.6)	7 (77.8)	56 (74.6)
NA	—	82 (8.4)		12 (1.4)		7 (1.5)		1 (0.5)		0 (0.0)

**Figure 1:**
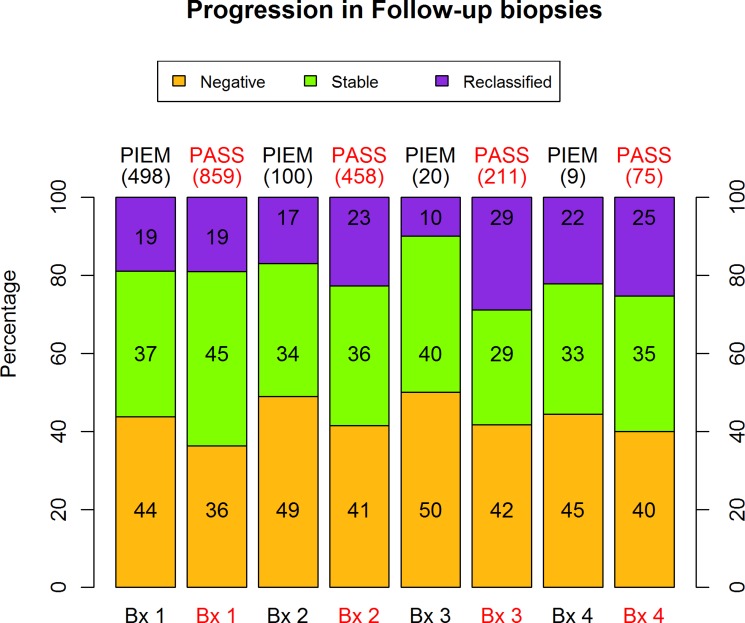
Percentage of reclassification in follow-up biopsies

The analysis of the variables that define reclassification shown in Figure 1 illustrates the percentages of reclassification for the PIEM and PASS database at the four follow-up biopsies. The calculated *p*-values of 0.92, 0.23, 0.12 and 0.99 respectively also reflect the equivalence between percentages of reclassification in all follow-up biopsies.

**Figure 2:**
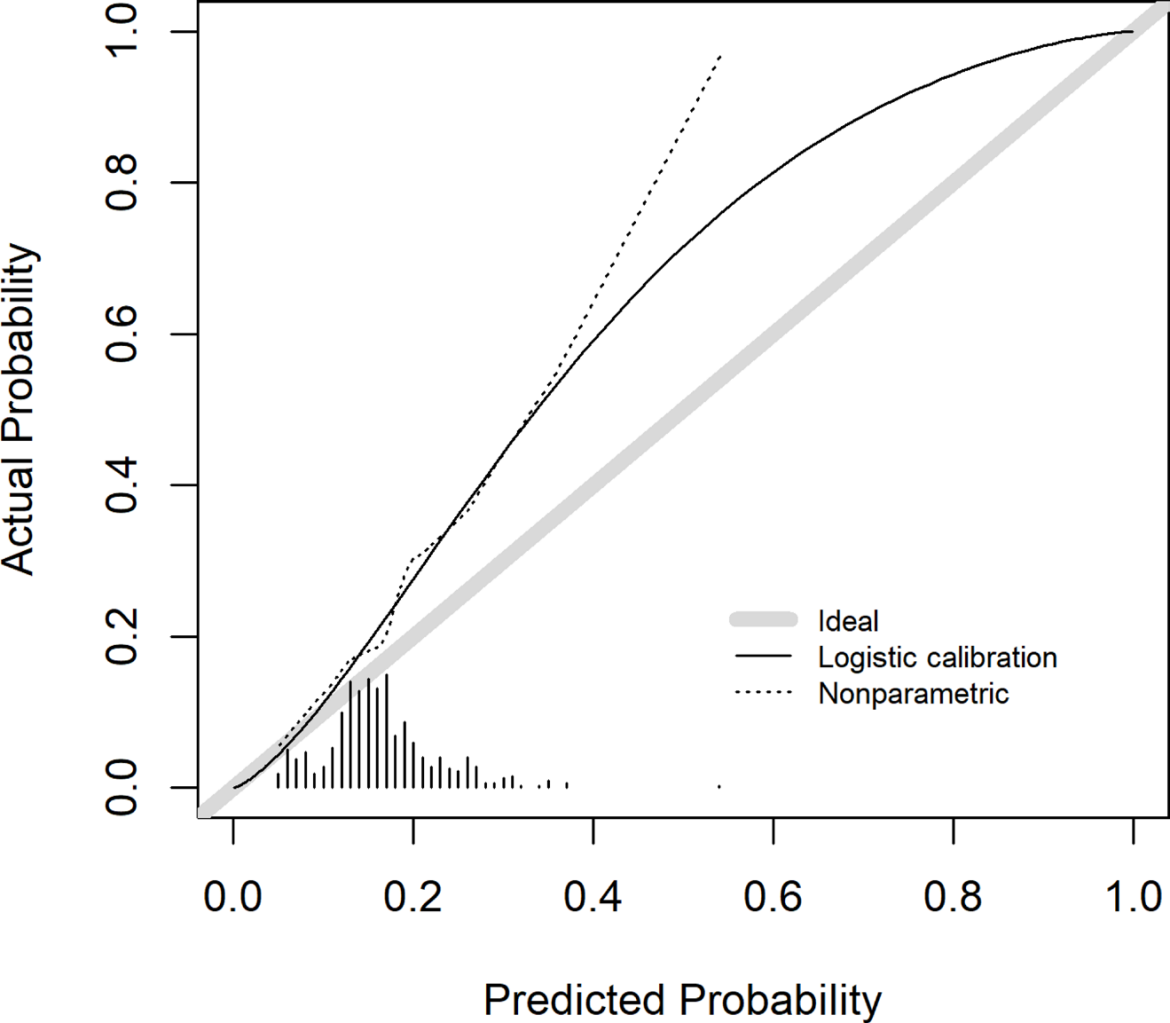
Calibration plot of PASS-RC validation in PIEM cohort

**Figure 3:**
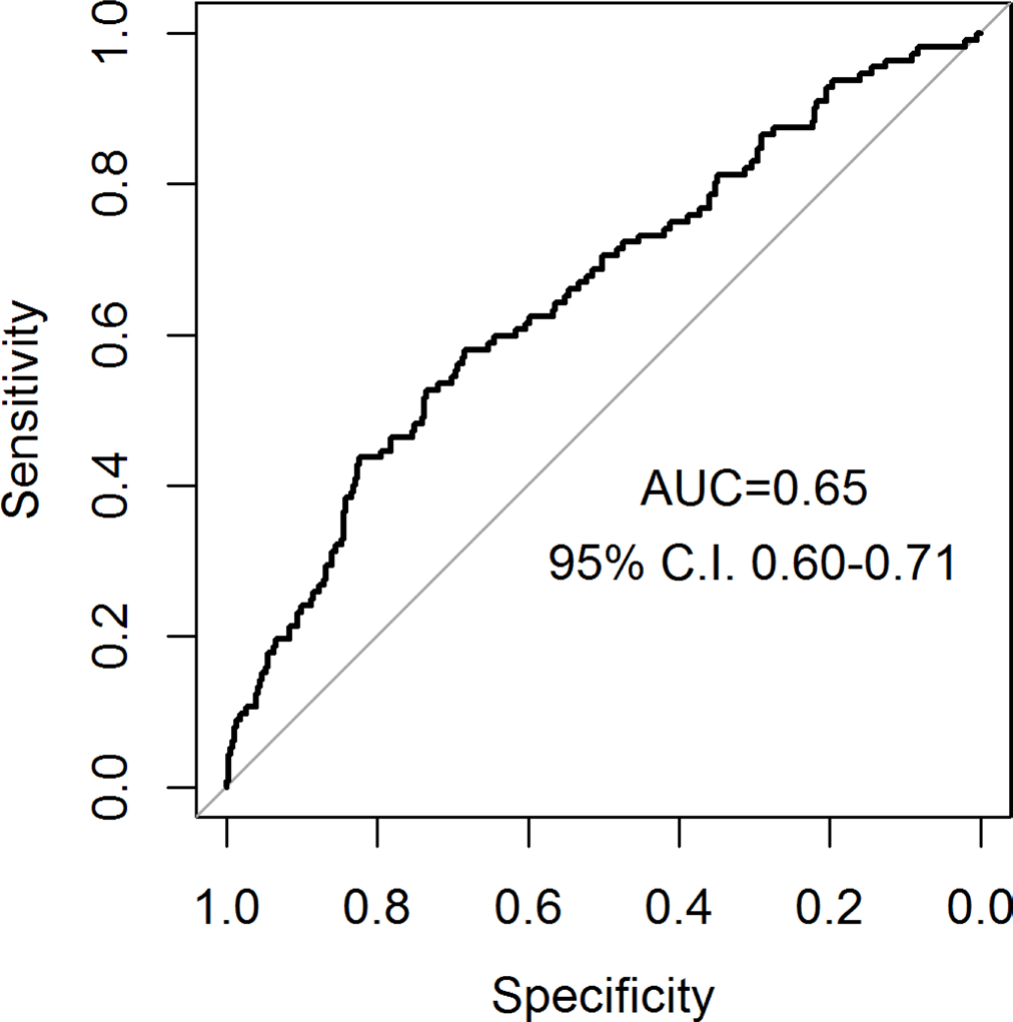
ROC curve of PASS-RC validation in PIEM cohort

As regards the validation of the PASS-RC, we obtained the calibration curve that shows an underestimation of reclassification probabilities (Figure 2). The ROC curve resulted in an area under the ROC curve (AUC) of 0.65, lower than the 0.72 obtained in the generation cohort [15] (Figure 3). No statistically significant differences appear in a comparison between Gleason 3+4 (AUC = 0.66) and Gleason 4+3 (AUC = 0.63) reclassifications, *p*-value = 0.55.

**Figure 4:**
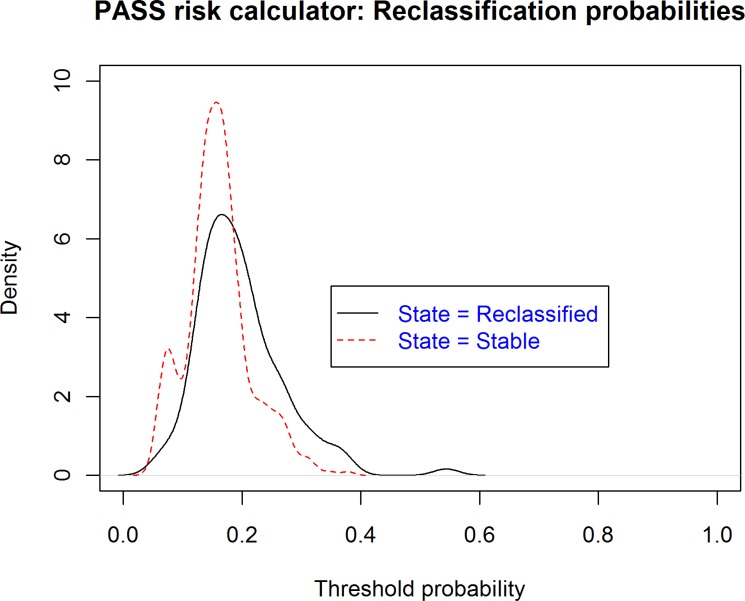
Probability density functions of probability values obtained from PASS-RC in patients with/without reclassification in PIEM cohort

**Figure 5:**
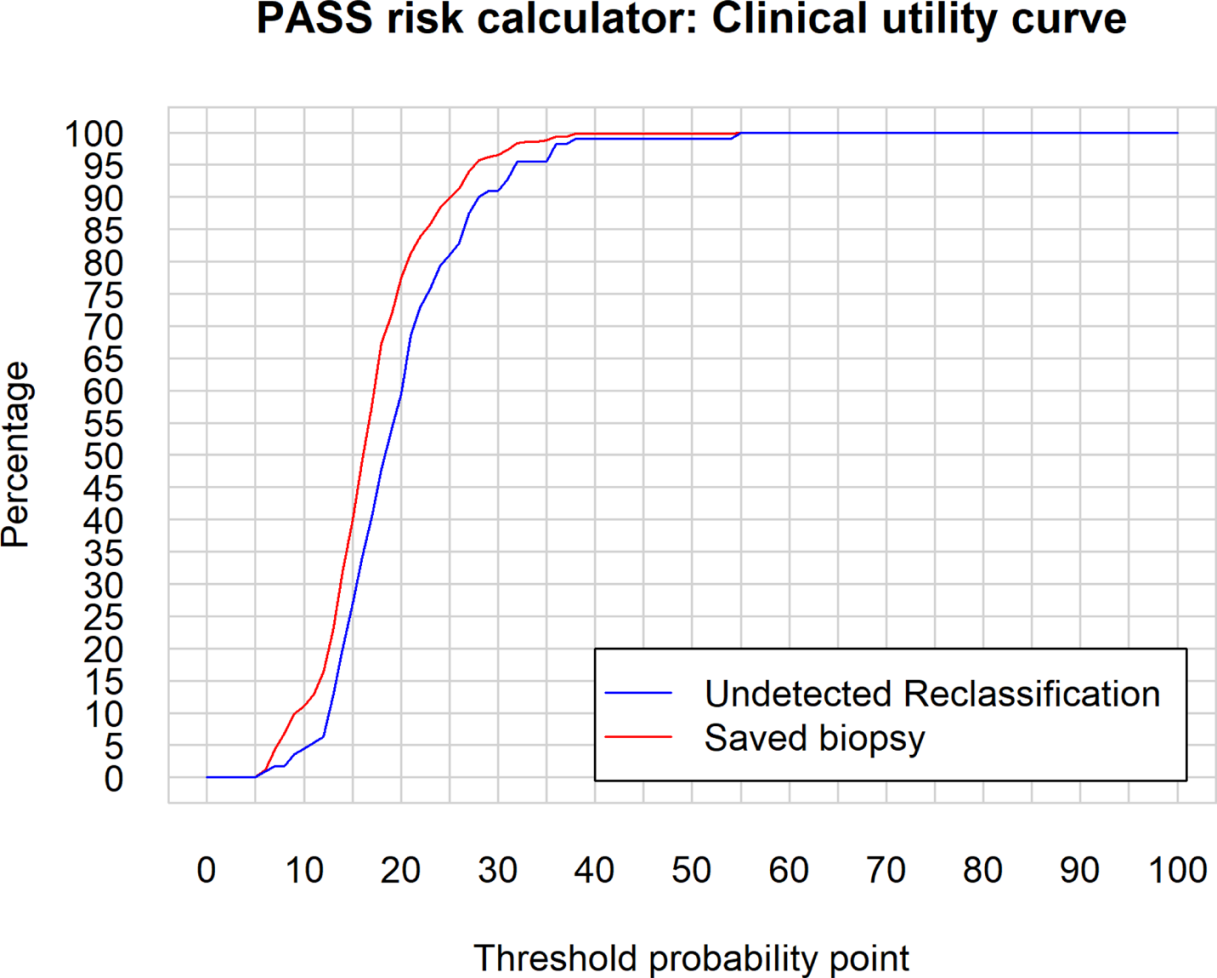
Clinical utility curve: For different threshold probability points selected in X axe, it can be seen in the Y axe, on the one hand, in blue line, the percentage of biopsies not performed to patients (Saved biopsy) and, in the other hand, in red line, the percentage of patients whose progression have been not been adequately diagnosed (Undetected reclassification)

**Figure 6:**
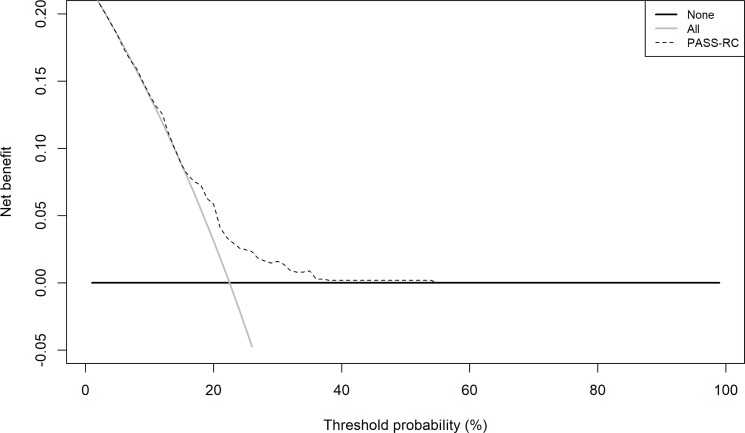
Decision curve analysis

Figure 4 shows the probability density function (PDFs) analysis performed. We can see the probabilities of reclassification provided by PASS-RC for the patients who progressed, or who did not, in the PIEM validation cohort, which proves the impossibility to identify a good discriminative cut-off to be recommended for clinical use. The Clinical utility curve (CUC), illustrated in Figure 5, highlights the real clinical impact of these findings. Selecting different threshold reclassification probabilities under those we should not indicate a follow-up Bx, we project this value over the two curves in the graphic. In one of them, the red one, we can see the percentage of saved follow up-Bx and in a parallel fashion, in the blue curve, the rate of missed reclassifications. Probably, the best threshold corresponds to 13%, which corresponds to a 23% of potentially saved biopsies at a cost of missing 13% reclassifications. Alternatively, in a more conservative way, a cut-off of 12%, drives us to a 16% of saved biopsies and a 6% of undetected reclassifications (Table 3, Figure 5). The decision curve, shown in Figure 6, assessed this analysis, there is a narrow range between 18%-38% where PASS-RC have utility, but with a poor net benefit below 10%.

**Table 3:** Clinical utility cut-offs analysis

Threshold (%)	Saved biopsies (%)	Undetected reclassifications (%)
5	0	0
6	1,21	0,9
7	4,4	1,8
8	6,8	1,8
9	9,9	3,6
10	11,1	4,5
11	12,9	5,4
12	16,3	6,3
13	22,7	12,6
14	31,8	19,8
15	40,0	27,0
16	49,3	34,2
17	57,7	40,5
18	67,4	47,7
19	71,8	54,0
20	77,5	59,5

In addition, we have explored the performance of the combination of PASS-RC with the predictors BMI, PSA density, PCa length in positive cores and the percentage of PCa involvement in those cores. Results are shown in Table 4. Only PSA density increase significantly the AUC from 0.654 to 0.694 ( *p* = 0.04).

**Table 4:** Combined models of PASS-RC analysis

	Reclassification			Reclassification		AUC test
	*p*-value	AUC		*p*-value	AUC	*p*-value
PASS-RC	< .001	0.654	PASS-RC+			
BMI^*^	0.041	0.577	BMI	0.047	0.653	0.429
PSAD	< .001	0.634	PSAD	< .001	0.694	0.043
Length^+^	0.192	0.551	Length	0.062	0.655	0.933
PCAi^++^	0.582	0.512	PCAi	0.792	0.549	0.691

BMI: body mass index. PSAD: PSA density. Length: PCa length in positive cores. PCAi: percentage of PCa involvement in biopsy. AUC: area under the ROC curve. BMI, Length and PCAi avalaible for ^*^n = 238, ^+^*n* = 171; ^++^*n* = 176.

## DISCUSSION

Active surveillance is becoming more and more common in our clinical practice. The unavoidable consequences of over-detection in PCa are not synonymous of over-treatment because AS contributes to a responsible management of patients in low or very low grade and low volume PCa [23]. Nevertheless, still many urologists are reluctant to offer AS to their patients [24], arguing a lack of confidence in conservative management instead of an active treatment, and due to uncertainties with their general pathologists and radiologists with no specific training in mpMRI, or lack of strong evidence that new biomarkers could help in this setting [23]. Other non scientific reasons could be explained by the need to justify economical and technical investments to their health care providers.

Above these arguments, we can mention the accumulative evidence of AS efficiency in PCa control of series with long follow up [22], showing cancer specific survival rates of 99.9% at 15 years with strictest criteria [25]. This strong argument minimizes the potential benefit in cancer specific survival of active treatment supporters. However, the serious implications of AS should be carefully explained to the patients. Given the urge for evolution, protocols are now trying to become risk-adapted. Yet, a follow-up Bx is not free of complications and it can become a clearly stressful episode and a source for discomfort that patients will need to confront. With this aim, protocols will need to be simplified due to the potential huge amount of AS candidates [26, 27], and the predicted costly work overload for Urology Departments.

Is in this context, the PASS initiative for developing an Active Surveillance Biopsy Risk Calculator of Reclassification/Upgrade should be considered as extremely convenient [15]. Previous studies have tried to associate PSA kinetics [28], number of previous positive cores in Bx and PSA density, or Gleason score and PSA at baseline [29] to assess the risk of reclassification. The PASS Risk Calculator (PASS-RC) calculates the individual risk of reclassification in the follow up Bx of patients on AS [14, 15]. The internal validation obtained an AUC-ROC of 0.72, which bears acceptable discrimination ability. The authors elegantly claim for external validations to test the real capacity of this Risk Calculator before recommending its general implementation [15].

In this study we have analyzed the usefulness of the PASS-RC in the Spanish Registry on AS, with 1,024 AS patients included in our Registry at December 2015. This registry was opened at September 2014 and patients were collected prospective and retrospectively from several Spanish series with more evolution on AS; but most of them have been included at the beginning of their evolution on AS. This is the reason because only 498 patients have at least one follow-up Bx for the present validation. This fact could be considered a limitation, but in our opinion the more relevant follow-up Bx on AS is the confirmation Bx, the Bx that certificate that the initial findings from the initial Bx are real and patients can continue on AS with a low risk of underclassification. So that we consider are validation focused on confirmation-Bx specially relevant to validate the clinical useful of PASS-RC. As mentioned, no standardized protocol of follow up is defined in our Registry, so each center uses its own protocol and defines its own cohort as a real life clinical practice on AS, offering PASS-RC the opportunity to show its real utility in common clinical practice. Statistically, the differences between reference and study cohorts (Table 1), rather than representing a drawback, offer PASS-RC an adequate opportunity for external validation. Thus, our series of 498 patients is older than the PASS cohort and shows higher PSA levels than the PASS cohort, mainly in the interval 6–10 ng/mL and it is more conservative in T stage at diagnosis.

Regarding the results of the first follow up Bx, no cancer was found in 43.8% of the cases, compared to 36% in the PASS cohort ( *p* < 0.01). In the following follow-up Bx this trend remained the same, without any significant difference. Reclassification rates during follow up Bx ranged between 10 and 22% in our series and between 20 and 30% in the reference series, without any statistical differences at any follow-up Bx between them (Figure 1). Therefore, we were able to unveil a trend to a lower reclassification rate in our series and a higher absence, or PCa, at our follow up Bx. To date we have been unable to assess if it would become statistically significant with a higher sample size in the future. Different diagnoses and AS strategies could have influenced these different outcomes. At the moment, the 2-month mean inferior interval between Bx in our cohort is the only objective justification for the lower detection of reclassification, particularly in cumulative follow-ups.

When we evaluated the correspondence between the predicted probabilities of the model in our validation series and the real incidence of reclassification, we observed how the model underestimates our reality of reclassification, mainly in the projection of high probabilities (Figure 2). In fact, the probabilities assigned by the model were low, ranging between 20 and 50% (Figures 2 and 4), are coherent with a series which has a low risk of reclassification as the AS series are. The discrimination ability was lower in our series (AUC: 0.65 (Figure 3), which is common in external validations but compromises the clinical utility of the model.

We also assessed the clinical utility of the model through probability density functions [30, 31]. We understand it is an excellent tool to choose cut-off points of clinical utility in predictive models, nomograms and risk calculators. Thus, in Figure 4 we can see the probabilities of reclassification provided by PASS-RC for both patients who progressed, or who did not, in the PIEM validation cohort. We expected higher probabilities of reclassification for patients who actually progressed but lower chances for the group of patients who did not.

From the distributions of probabilities analyzed, we tried to identify a cut-off which separated non-progressive patients (ideally, those with low probabilities assigned by the model and being under this cut-off) from true-progressive patients (with assigned probabilities over this cut-off point), but we were not able to obtain conclusive results. Unfortunately, the assigned probabilities are extremely similar in their distributions between both subpopulations without the opportunity to identify a discriminative cut-off point (Figure 4).

Finally, in an attempt to maximize the evaluation of this PASS-RC tool we drew our proposal of clinical utility curves as previously [31] (Figure 5). We have shown how the range of probabilities for clinical decisions rises from 5% to 40%. If we decided not to practice a follow-up Bx to patients with a reclassification probability under 13%, we would save 23% of preplanned Bx, but would fail to detect 13% of the progressive patients in our common clinical practice of follow-up Bx, dangerous percentage of missing progressive patients especially in protocols where the follow-up biopsies are preplanned every three years after the confirmatory Bx.

We must acknowledge two main limitations in this paper. Firstly, the multicentricity and the lack of a standardized protocol of AS management in our entire cohort is a fruitful opportunity to externally validate PASS-RC in common clinical practice, but could have driven to the underdetection or misclassification of reclassification. A short follow-up can be mentioned as the second limitation of our study and more specifically the small sample size included in the last biopsies.

It is now clear that other known series of AS should validate the PASS-RC to confirm our findings, given that these clinical tools [32–33] are widely used in daily practice. As the authors suggest in their publication, we do agree that these tools have to be refined with time, using longer follow-up, and if possible, using the same common inexpensive variables. But they will probably be improved with new biomarkers [34–36] or morpho-functional parameters from mpMRI, as shown in other models [37]. In this sense, we have verified the improvement provided by PSA density. We strongly encourage the evaluation of this kind of clinical predictive tools – nomograms and risk calculators – using our proposed probability density functions and clinical utility curves, both in generation and validation cohorts. These graphs show the real classificatory accuracy of their predictions and help us to choose the best cut-off points for clinical use.

## CONCLUSIONS

Using the Spanish multicentric registry study on AS as a validation cohort, we have obtained a moderate discrimination ability of PASS-RC but we found that it is not possible to choose a useful cut-off point to made adequate decisions in our clinical practice. Other external validations, the inclusion of new biomarkers, and especially the addition of morpho-functional parameters from mpMRI, could be implemented in future investigations to improve this model or to generate new ones.

## MATERIALS AND METHODS

In the present study, we validate the PASS-RC as a predictive tool of the reclassification on follow-up Bx for patients on AS with one independent external series extracted from the AEU/PIEM/2014/0001 registry. We retrospectively and prospectively collected data from 1,024 patients in a multicentric study which included 36 Spanish hospitals.

The inclusion criteria were the same as those used for the PASS, namely a Gleason score ≤6 and at least 10 cores on the initial Bx. In addition, the initial PSA value was below or equal to 20 ng/ml. No unique follow-up protocol was determined for the AEU series, so every center scheduled their own AS protocol and it does not necessarily coincide with the PASS protocol. The outcome of our validation was reclassification (or disease upgrade), defined by the PASS-RC as either Gleason score upgrade from ≤6 to ≥7 and/or as an increase in percentage of cancer cores positive for cancer from <34% to ≥34%. A comparative descriptive analysis between PIEM and PASS cohorts at diagnosis was performed. In order to compare both cohorts, the age and PSA variables were categorized and statistical significant differences were calculated using chi-squared test (Table 1). We also included a comparison between both series regarding the data of the follow-up Bx (Table 2).

We evaluated the predictive accuracy of the PASS-RC among our patients performing a standardized validation. Firstly, we obtained the probabilities estimated in the PIEM cohort using the PASS-RC [14, 15]. We drew the calibration curves, which evaluated the correspondence between the predicted and the actual probabilities of reclassification found in our cohort. Then, to study discrimination ability and the clinical utility of the model, the empirical distributions of probabilities of reclassification in the groups which actually reclassifies or not were graphically shown as the probability density functions (PDF) by kernel density estimation [38]. The overlap of the probability distributions of both populations was important to determine how the model discriminates between groups with and without the evaluated event. It also shows whether it was possible to choose a threshold for clinical application in order to split risk groups for reclassification. We proposed this PDF as a very useful way to choose the probability thresholds of clinical utility in previous validations of prostate cancer estimations [30, 31]. Moreover, discrimination was also quantified through the Receiver Operating Characteristics (ROC) curve [39], the area under the ROC curve (AUC) and its 95% confidence interval (CI). In order to estimate the saved biopsies and the reclassification delayed for different probabilities thresholds, we performed a graphical analysis through the Clinical utility curve (CUC) that we had previously designed to help this kind of clinical decisions [31]. Decision curve analysis was used to confirm the clinical utility analysis.

In addition, we explored, as other authors in previous studies [40, 41], the variables Body Mass index (BMI), PSA density, PCa length in positive cores and the percentage of PCa involvement as predictors of reclassifications, but here, in order to improve the discriminatory ability of PASS-RC. The AUC of PASS-RC and the combination of the variables with PASS-RC were compared using the DeLong Test [42].

Statistical analyses were performed at the two-sided 0.05 significance level, using R programming language v.3.2.1 [43].
